# Interfacial Engineering of PVDF‐TrFE toward Higher Piezoelectric, Ferroelectric, and Dielectric Performance for Sensing and Energy Harvesting Applications

**DOI:** 10.1002/advs.202205942

**Published:** 2023-01-03

**Authors:** Hamed Abdolmaleki, Astri Bjørnetun Haugen, Kristian Birk Buhl, Kim Daasbjerg, Shweta Agarwala

**Affiliations:** ^1^ Department of Electrical and Computer Engineering Aarhus University Aarhus Denmark; ^2^ Department of Energy Conversion and Storage Technical University of Denmark (DTU) Lyngby Denmark; ^3^ Danish Graphene ApS Vejle Denmark; ^4^ Novo Nordisk Foundation (NNF) Research Center Department of Chemistry and Interdisciplinary Nanoscience Center (iNANO) Aarhus University Aarhus Denmark

**Keywords:** 2D materials, energy harvester, flexible electronics, organic electronics, pressure sensor, PVDF‐TrFE

## Abstract

The electrical properties of pristine fluoropolymers are inferior due to their low polar crystalline phase content and rigid dipoles that tend to retain their fixed moment and orientation. Several strategies, such as electrospinning, electrohydrodynamic pulling, and template‐assisted growing, have been proven to enhance the electrical properties of fluoropolymers; however, these techniques are mostly very hard to scale‐up and expensive. Here, a facile interfacial engineering approach based on amine‐functionalized graphene oxide (AGO) is proposed to manipulate the intermolecular interactions in poly(vinylidenefluoride‐trifluoroethylene) (PVDF‐TrFE) to induce *β*‐phase formation, enlarge the lamellae dimensions, and align the micro‐dipoles. The coexistence of primary amine and hydroxyl groups on AGO nanosheets offers strong hydrogen bonding with fluorine atoms, which facilitates domain alignment, resulting in an exceptional remnant polarization of 11.3 µC cm^−2^. PVDF‐TrFE films with 0.1 wt.% AGO demonstrate voltage coefficient, energy density, and energy‐harvesting figure of merit values of 0.30 Vm N^−1^, 4.75 J cm^−3^, and 14 pm^3^ J^−1^, respectively, making it outstanding compared with state‐of‐the‐art ceramic‐free ferroelectric films. It is believed that this work can open‐up new insights toward structural and morphological tailoring of fluoropolymers to enhance their electrical and electromechanical performance and pave the way for their industrial deployment in next‐generation wearables and human‐machine interfaces.

## Graphical abstract

In this research, a facile interfacial engineering approach based on amine‐functionalized graphene oxide (AGO) is presented to improve the electrical properties of fluoropolymers. It is shown that AGO incorporation can lead to a higher *β*/*α* ratio and larger lamellae dimensions, which is translated into higher remnant polarization, and larger longitudinal piezoelectric coefficient in PVDF‐TrFE.

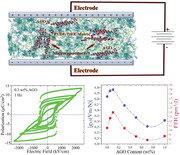

## 1 Introduction

As the current global energy crisis and chip shortage continues, the importance of switching to sustainable energy sources and developing alternative electronic materials is becoming more and more pronounced. Development of low‐power electronics in the last few decades has led to the possibility of using new energy technologies that harvest ambient energies to power the devices.^[^ ^1^ ^]^ Piezoelectric materials are promising candidates in this regard, as they can convert mechanical energy from various sources such as human motion, vibration, acoustic wave, etc., into electricity. For many years, lead zirconate titanate (PZT) has been the main material employed in piezoelectric devices; however, due to the toxicity, brittleness, and high relative permittivity, a great deal of research are being dedicated to replace it with flexible and biocompatible alternatives.

Polyvinylidene fluoride‐trifluoroethylene (PVDF‐TrFE) is a versatile piezoelectric polymer with ferroelectric and pyroelectric properties, which has been employed in energy harvesters, sensors, film capacitors, batteries, transistors, non‐volatile random‐access memories, acoustic resonators, ultrasonic transducers, and tissue engineering. Despite showing promising results, there are some drawbacks associated with its device performance. For instance, in terms of dielectric behavior, the high energy density obtained from PVDF‐TrFE is normally accompanied with a high loss factor and leakage current due to the ferroelectric nature of the polymer, resulting in poor charge–discharge efficiency when used in energy storage systems.^[^ ^2^ ^]^ In terms of longitudinal piezoelectric coefficient (*d*_33_), PVDF‐TrFE is one the best organic piezoelectrics (|*d*_33_| ≈ 30–40 pC N^−1^); however, when compared with commercial piezoceramics such as PZT (*d*_33_ ≈ 200–600 pC N^−1^) and BaTiO_3_ (*d*_33_ ≈ 200 pC N^−1^) it is still inferior.^[^ ^3^ ^]^ Furthermore, the negative nature of the piezoelectric effect in PVDF‐TrFE makes it hard to boost its performance by incorporating piezoceramics, where the *d*_33_ is positive. Despite lower *d*_33_, PVDF‐TrFE is a superior candidate for voltage‐type applications such as piezoelectric sensors due to the high voltage coefficient (*g*_33_ ≈ 0.3 Vm N^−1^) emanating from its low relative permittivity. Moreover, high figure of merit for non‐resonant energy harvesting (*d*_33_.*g*_33_), low acoustic impedance, which matches that of human tissues, along with flexibility and biocompatibility, makes PVDF‐TrFE an exceptional choice for wearable energy harvester and human‐machine interfaces.^[^ ^4^ ^]^

The ferroelectric properties of PVDF‐TrFE have been investigated in ferroelectric random‐access memories (FRAM). Compared to dynamic and static random‐access memories (DRAM and SRAM), FRAM has the advantage of being non‐volatile, as it does not require a power supply to maintain stored data. They also offer faster write and read time, higher endurance, and lower energy consumption compared to conventional non‐volatile Flash and electronically erasable programmable read‐only memories (EEPROM).^[^ ^5^ ^]^ For FRAM applications, ferroelectric materials should demonstrate a high remnant polarization (*P*_r_) and low coercive field (*E*_c_). These values for PVDF‐TrFE are inferior to PZT, which is the most widely used material in FRAMs, thus, a huge room for improvement still exists.

The electrical properties of PVDF‐TrFE are structure dependent and can be tailored by modifying the degree of crystallinity, ratio of crystalline phases, crystallite size, and morphology. These objectives are typically pursued by either processing or composite strategies.^[^ ^6^ ^]^ The processing strategies are practices followed during or after the production stage to alter crystallization mechanism and morphological structures of the polymer. Well‐known examples are electrospinning,^[^ ^7^ ^]^ electrohydrodynamic pulling,^[^ ^8^ ^]^ template‐assisted growing,^[^ ^9^ ^]^ thermal annealing,^[^ ^10^ ^]^ electric polling,^[^ ^11^ ^]^ corona discharge polling,^[^ ^12^ ^]^ and mechanical stretching.^[^ ^13^ ^]^ On the other hand, composite strategies involve addition of functional fillers into polymer matrix to influence the crystallinity and nanostructures, and/or endow certain electrical properties. In terms of filler loading, composites can be classified into two categories, i.e., composites with low or ultralow filler content (<1 wt.%) and composites with medium to high filler content (>1 wt.%). The idea behind employing medium to high filler content is mainly to incorporate the superior electrical properties of fillers into the polymer matrix, examples are, addition of conductive fillers up to the percolation threshold^[^ ^14^ ^]^ or introducing a high percentage of ceramic nanoparticles into the polymer matrix to boost the dielectric performance. Utilizing low and ultralow filler ratio is typically to modify the crystallinity, nanostructures, and forming new interfaces,^[^ ^15^ ^]^ such as incorporation of reduced graphene oxide (rGO)^[^ ^16^ ^]^ or Ti_3_C_2_T*_x_* MXene^[^ ^17^ ^]^ into fluoropolymers to boost their piezoelectric performance.

In this work, we propose an interfacial engineering approach based on amine‐functionalized graphene oxide (AGO) to manipulate the intermolecular interactions in (PVDF‐TrFE) to induce *β*‐phase formation, enlarge the lamellae dimensions, and align the micro‐dipoles. The main reason for using AGO is the electron‐rich nature of primary amine groups, which can reduce the zeta potential and form stronger electrostatic intermolecular interactions with polar C—F bonds, which leads to outstanding improvement in piezoelectric properties. Moreover, it can also improve the net polarization and dielectric behavior of the nanocomposites due to its large dipole moment. The influence of AGO on remnant polarization, coercive field, longitudinal piezoelectric coefficient, hysteresis behavior, relative permittivity, dielectric breakdown strength, loss tangent, and leakage current of PVDF‐TrFE films is investigated comprehensively. Furthermore, by using calorimetry methods, wide angle X‐ray scattering (WAXS), and small angle X‐ray scattering (SAXS) techniques, the correlation of the crystallinity, nanostructures, and lamellae dimensions, with electrical performance of the nanocomposite films is elucidated. The nanocomposite film with 0.1 wt.% AGO demonstrated superior piezoelectric and ferroelectric performance compared with state‐of‐the‐art reports in the literature.^[^ ^18^ ^]^ Recent reports with other carbon allotropes and 2D materials can be found in the following references.^[^ ^19^ ^]^

## 2 Results and Discussion

### 2.1 Synthesis and Characterization of AGO

**Figure 1:**
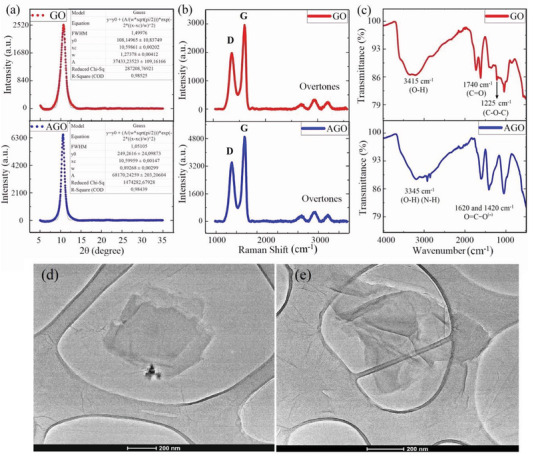
Structural characterization of AGO. a) X‐ray diffraction, b) Raman, and c) FT‐IR spectra of synthesized GO and AGO. d,e) TEM images of AGO nanosheets

XRD analysis was used to investigate the crystal structure of synthesized GO and AGO (**Figure** **1**a). As seen, both samples demonstrated a sharp diffraction peak at 2*θ* = 10.59°, corresponding to the (100) planes. The crystallite size of GO and AGO was found to be 5.56 and 7.93 nm, respectively, calculated by inserting full width at half‐maximum (FWHM) values of the diffraction peaks in the Scherrer equation. The Raman spectra of the samples demonstrated two strong peaks at 1,356 and 1,600 cm^−1^, corresponding to the D and G bands, respectively (Figure 1b). The D band originates from coupling between electrons and A_1g_ phonons near the K point of the Brillouin zone and corresponds to the local defects and disorders in graphitic structures, while the G band stems from the E_2g_ phonon mode of C=C graphitic structures.^[^ ^20^ ^]^ Three small peaks located at 2,700, 2,930, and 3,195 cm^−1^ are the overtone bands of 2D, D + G, and 2G, respectively, arising from the vibrational transitions with Δ*v* *=* 2. Both Raman spectra are nearly identical with *I*_D_ */I*_G_ of 0.67 and 0.69 for GO and AGO, corroborating that the amination process neither introduced new defects nor changed the density of delocalized electrons (*I*_D_ and *I*_G_ are the intensity of D and G peaks, respectively).

The FT‐IR spectrum of GO confirmed the presence of C—O (1,044 cm^−1^), C—O—C (1,225 cm^−1^), C—OH (1,414 cm^−1^), and carboxylic C=O (1,740 cm^−1^) (Figure 1c), while that of AGO demonstrated two peaks at 1,620 and 1,420 cm^−1^, corresponding to the symmetric and asymmetric C—O stretches of carboxylate groups. The change in absorption pattern going from carboxylic for GO to carboxylate for AGO indicates that at least a portion of the introduced ammonia during the amination process has been consumed to convert carboxylic acid groups to ammonium carboxylates. Furthermore, the fact that the peak corresponding to the epoxide groups at 1,225 cm^−1^ is no longer present in the AGO spectrum, suggests an amination reaction through epoxide ring opening. Transmission electron microscopy (TEM) images of AGO nanosheets are demonstrated in Figure 1d,e. The images confirmed the formation of few layered AGO nanosheets with different lateral dimensions. Scanning electron microscopy (SEM) technique was used to determine the mean lateral area of the synthesized AGO nanosheets (Figure S1c, Supporting Information). By measuring the lateral area of 100 discrete nanosheets, the mean area was obtained to be 0.14 ± 0.03 µm^2^.

**Figure 2:**
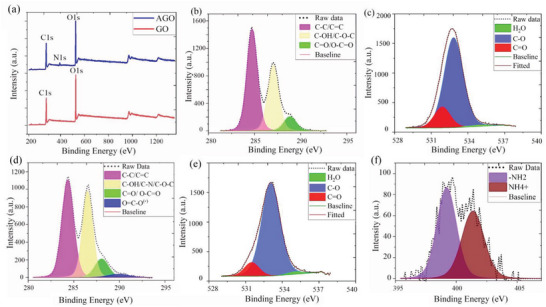
XPS analysis of AGO. a) XPS survey spectra of GO and AGO. b) Deconvoluted high‐resolution C1s spectrum of GO. c) Deconvoluted high‐resolution O1s spectrum of GO. d) Deconvoluted high‐resolution C1s spectrum of AGO. e) Deconvoluted high‐resolution O1s spectrum of AGO. f) Deconvoluted high‐resolution N1s spectrum of AGO.

To confirm the hypothesis regarding the epoxide ring opening reaction, high‐resolution X‐ray photoelectron spectroscopy (XPS) analysis was carried out on GO and AGO samples. The XPS survey spectra demonstrated that GO is comprised of 71.3 ± 1.1% carbon and 27.3 ± 1.1% oxygen, while, AGO had 67.4 ± 1.7% carbon, 28.7 ± 1.3% oxygen, and 3.4 ± 0.4% nitrogen (**Figure** **2**a; Table S1, Supporting Information). The high‐resolution C1s spectrum of GO was deconvoluted into three peaks at 284.6, 287.0, and 288.8 eV corresponding to C—C/C=C (52.7 ± 3.6%), C—OH/C—O—C (38.9 ± 2.9%), and C=O/O—C=O (8.4 ± 1.0%), respectively (Figure 2b; Table S2, Supporting Information).^[^ ^21^ ^]^ In comparison, the C1s deconvolution of AGO resolved into four peaks at 284.4 (47.3 ± 2.7%), 286.6 (37.0 ± 2.7%), 288.0 (13.4 ± 1.5%), and 290.3 eV (2.3 ± 0.7%), where the newly formed peak at 290.3 eV was attributed to the carboxylate groups with ammonium counter ions (Figure 2d; Table S3, Supporting Information). The redshift from 287.0 to 286.6 eV in the C—OH/C—O—C peak would seem to confirm the conversion of most epoxide groups to HO‐CR_2_‐CR_2_‐NH_2_,^[^ ^22^ ^]^ as the newly formed C—N bonds have lower binding energy compared to C—O. The high‐resolution O1s spectra of GO and AGO nanosheets are shown in Figure 2c,e, respectively. Both graphs were deconvoluted into 3 peaks corresponding to C—O (533.0 eV), C=O (532.0 and 531.5 eV), and residual H_2_O (535.0 eV). The shift in the position of C=O bonds from 532.0 eV for GO to 531.5 eV for AGO can be attributed to conversion of carboxylic groups to carboxylates. The high‐resolution N1s spectrum of AGO further confirmed the epoxide ring‐opening hypothesis by demonstrating two peaks at 399.1 and 401.2 eV, attributed to primary amines and ammonium ions, respectively (Figure 2d). In summary, it was concluded that introduced ammonia reacted with GO in two parallel ways, one by reacting with carboxylic acid groups to form ammonium carboxylate, and the other by opening the epoxide groups to form primary amines.

### 2.2 Crystallinity and Structural Characterization

**Figure 3:**
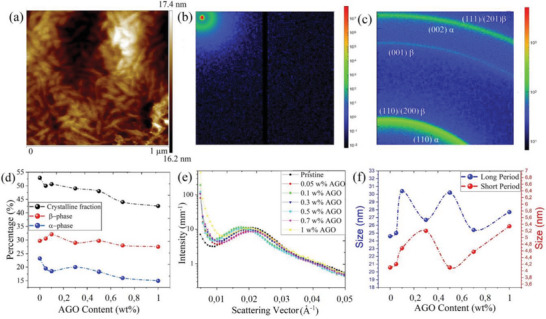
Crystallinity analyses of PVDF‐TrFE and AGO nanocomposite films. a) AFM image of 0.1 wt.% AGO nanocomposite film. b) 2D‐SAXS pattern for PVDF‐TrFE. c) 2D‐WAXS pattern for PVDF‐TrFE. d) Total degree of crystallinity and fraction of each crystalline phases obtained from DSC and WAXS analyses. e) 1D SAXS plots for the pristine PVDF‐TrFE and the AGO nanocomposite films. f) Long‐period and short‐period parameters obtained from Strobl model.

AFM studies demonstrated stacked edge‐on lamellae crystallites surrounded by amorphous matrix in the pristine PVDF‐TrFE and the AGO nanocomposite films, resulting in randomly oriented needle‐like crystalline domains (**Figure** **3**a; Figure S2, Supporting Information). The 2D SAXS scattering pattern and the well‐defined ring structure obtained from 2D‐WAXS also implied isotropic orientation and distribution of crystallites within the films (Figure 3b,c; Figures S3 and S4, Supporting Information). When fillers interact with semicrystalline polymers during nucleation and lamellae growth stage, changes in micro and nanostructures are likely to happen in multiple ways. Some possible scenarios can be: I) fillers acting as nucleating agents, thus reducing the overall Gibb´s free energy of embryo formation; II) fillers favoring a particular crystalline phase over others due to certain zeta potential and surface energy; III) fillers inhibiting crystallization via steric hindrance and/or strong intermolecular interaction with polymer; and IV) fillers altering the lamellae dimensions and orientation. To investigate the impact of AGO incorporation on crystallinity and nanostructures of PVDF‐TrFE, a comprehensive study based on DSC, SAXS, and WAXS analyses was carried out (Figure 3b–f). The results demonstrated the presence of two crystalline phases with trans‐gauche (TGTG) and all‐trans (TTTT) conformations within an amorphous matrix in both pristine PVDF‐TrFE and the AGO nanocomposite films. The calorimetry data signified a gradual decrease in the total degree of crystallinity (*χ*_*c*_) by increasing the AGO content, which led to a decrease in *χ*_*c*_ from 52.9% for the pristine polymer to 42.7% for the 1 wt.% AGO nanocomposite. The nanocomposite film with 0.1 wt.% AGO experienced the lowest decline in the crystallinity with *χ*_*c*_= 50.6% (Figure 3d; Figure S5, Supporting Information).

WAXS analysis demonstrated that AGO incorporation has a significant impact on the ratio of *β*/*α* crystalline phases. Briefly, all the nanocomposite films had a higher *β*/*α* ratio compared with the pristine polymer, and this ratio increased with increasing the AGO concentration. The pristine PVDF‐TrFE had *β*/*α* = 1.27, while this ratio for the nanocomposite films with 0.1 and 1 wt.% AGO was 1.73 and 1.83, respectively, which corroborates a higher thermodynamic favorability of the polar *β* phase formation in the presence of AGO. This phenomenon is of great importance as the piezoelectric and ferroelectric properties of PVDF‐TrFE mainly originate from non‐centrosymmetric *β* phase. Considering the decrease in *χ*_*c*_, the nanocomposite film with 0.1 wt.% AGO had the highest overall *β* phase content of 32.1% (Figure 3d).

Electrical and mechanical properties of semicrystalline polymers like PVDF‐TrFE are also influenced by the lamellae structure and crystalline‐amorphous interfaces. SAXS analysis revealed that the lamellae spacing (*d*) increases with addition of AGO, but not in a linear manner. The value of *d* for the pristine PVDF‐TrFE film was 29.9 nm, while that for the nanocomposites with 0.1 and 0.5 wt.% AGO was 36.9 and 35.1 nm, respectively (Figure 3e). Although *d* indicates the average distance between lamellae, it is uninformative about the thickness proportion of amorphous and crystalline regions in a domain. To further investigate the nano‐morphology of the samples, electron density auto‐correlation function of density fluctuation *K(z)*, was obtained from the raw SAXS data.^[^ ^23^ ^]^ By using the model developed by Strobl and Schneider the long period (*L*_p_) and short period (*S*_p_) parameters of the lamellae structure were obtained (Figure 3f; Figure S6, Supporting Information).^[^ ^24^ ^]^ As can be observed, all the nanocomposite films demonstrated larger *L*_p_ compared to the pristine polymer, with the largest value found for the 0.1 wt.% AGO nanocomposite (*L*_p_ *=* 30.4). Likewise, the *S*_p_ value for the nanocomposite films was also higher or equal to that for pristine polymer, indicating that thicker amorphous blocks surrounding the crystallites were formed. By subtracting values of *S*_p_ from *L*_p_, the length of the crystallites was calculated. Pristine PVDF‐TrFE possessed the smallest crystallite size (20.5 nm), while the 0.5 and 0.1 wt.% AGO nanocomposite films formed the biggest crystallites (26.1 and 25.7 nm, respectively). Briefly, from these results it can be inferred that the presence of AGO led to a lower rate of nucleation, while facilitating crystal growth, which in turn, resulted in the formation of bigger crystallites.

### 2.3 Dielectric Performance

The maximum possible stored energy in a dielectric capacitor (*U*_max_) is proportional to its permittivity (*ε*_0_ *ε*_*r*_) and dielectric breakdown strength (*E*_b_) (Equation 1); therefore, simultaneous enhancement of those properties is the key step toward developing more powerful energy storage systems.

(1) $$U_{max}=\;\int_{0}^{E_{b}}\varepsilon_{0}\varepsilon_{r}EdE$$

**Figure 4:**
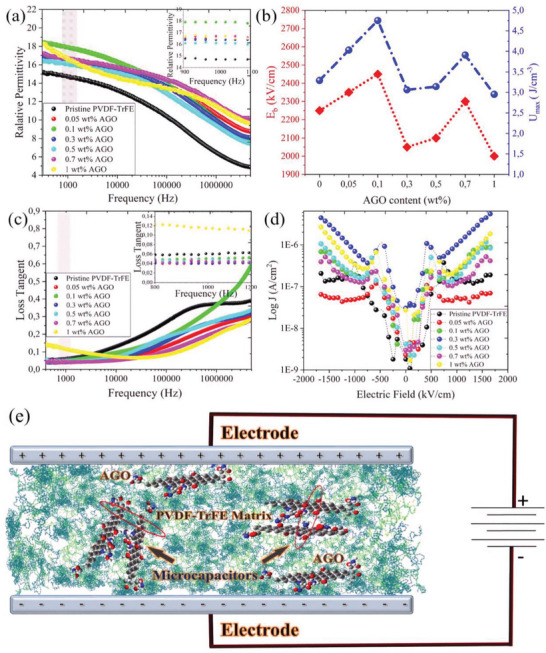
Dielectric characterization of PVDF‐TrFE and the AGO nanocomposites. a) Frequency‐dependent relative permittivity, b) dielectric breakdown strength and energy density, c) frequency‐dependent loss tangent, and d) field‐dependent leakage current density of the pristine PVDF‐TrFE and the AGO nanocomposite films. e) Schematic of formation of microcapacitors within an AGO nanocomposite film capacitor.

**Figure 5:**
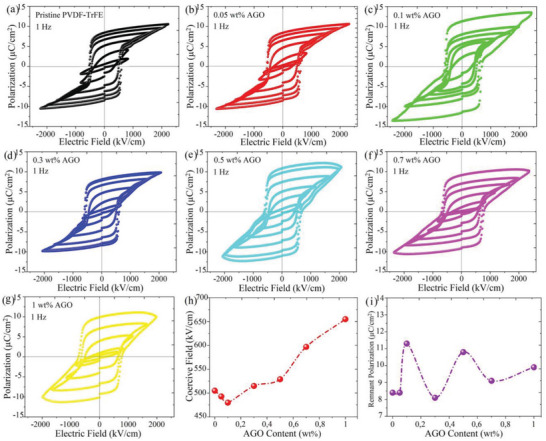
Ferroelectric characterization of PVDF‐TrFE and the AGO nanocomposites. P‐E hysteresis loops of a) pristine PVDF‐TrFE, b) 0.05 wt.% AGO nanocomposite, c) 0.1 wt.% AGO nanocomposite, d) 0.3 wt.% AGO nanocomposite, e) 0.5 wt.% AGO nanocomposite, f) 0.7 wt.% AGO nanocomposite, and g) 1 wt.% AGO nanocomposite. h) Coercive field values for pristine PVDF‐TrFE and AGO nanocomposites at an applied electric field of 1,166 kV cm^−1^. i) Remnant polarization of pristine PVDF‐TrFE and the AGO nanocomposites obtained at the highest field strength.

**Figure** **4**a shows the relative permittivity (*ε*_*r*_) of the pristine PVDF‐TrFE and the AGO nanocomposite films in the frequency range from 300 Hz to 5 MHz. All the samples demonstrated a notable decrease in the relative permittivity as frequency increases, which is attributed to the reduction of the space charge polarization effect (known as interfacial polarization).^[^ ^25^ ^]^ As can be observed, an improvement in the relative permittivity of all the AGO nanocomposites is notable within the whole frequency range, when compared to the pristine PVDF‐TrFE. At 1 kHz, the 0.1 wt.% AGO nanocomposite film had the highest relative permittivity of *ε*_r_ = 17.9, being 22% higher than that of the pristine PVDF‐TrFE. This enhancement can be explained by microcapacitor effect, in which the electron‐rich sp^2^ hybridized structures in AGO are surrounded by thin polymer chains to form numerous microcapacitor structures within the dielectric layer.^[^ ^26^ ^]^ The nonlinear increase in the relative permittivity with increasing the AGO loading might be due to AGO aggregation at higher concentrations and also approaching the percolation threshold. In addition, presence of C‐NH_2_ moieties with a highly polarizable permanent dipole moment can contribute to the permittivity enhancement in the AGO nanocomposite films. **Figure** **5**b shows the values of dielectric breakdown strength. The 0.1 wt.% AGO nanocomposite film demonstrated a pronounced increase in the dielectric breakdown strength (*E*_b_ = 2450 kV cm^−1^), which was 200 kV cm^−1^ higher than that of the pristine polymer. By inserting the obtained permittivity and *E*_b_ values into the Equation 1, the *U*_max_ value for each sample was calculated (Figure 4b). Briefly, the highest stored energy per unit volume was obtained in the 0.1 wt.% AGO film with *U*_max_ = 4.75 J cm^−3^, which was 30% higher than that of pristine PVDF‐TrFE. This value is also significantly higher than the *U*_max_ for state‐of‐the‐art paraelectric polymers used in commercial film capacitors like biaxially oriented polypropylene (BOPP), which is typically below 2 J cm^−3^.^[^ ^27^ ^]^ The working mechanism of the PVDF‐TrFE – AGO nanocomposite film capacitors is based on the fact that the energy bandgap of the polymer is too high^[^ ^28^ ^]^ (≈5–6 eV), so that the electric field cannot excite the electrons from its valance band into the conduction band, thus cannot cause the charge carrier flow through the film. Instead, the electric field results in electric polarization of the dielectric material by displacement of the positive charges toward the electric field and the negative charges opposite to the electric field. This charge separation serves as a potential electric energy in the film capacitor.^[^ ^29^ ^][^ ^30^ ^]^

Most ferroelectric polymers demonstrate a higher relative permittivity compared to paraelectric polymers but their energy loss factors are also significantly higher.^[^ ^31^ ^]^ Generally, there are two forms of energy loss phenomena within insulators. One is dielectric loss (parametrized as loss tangent or *tan δ*), which is caused by dissipation of energy in the form of heat, and the other is conduction loss, which is emanated from the flow of charge carriers via mechanisms such as hopping, tunneling, Schottky emission, Poole–Frenkel emission, etc., and is parametrized as leakage current.^[^ ^32^ ^]^ High dielectric loss in ferroelectric polymers is intrinsic to some extent and mainly originates from their ability to consume electromagnetic energy for internal dipole alignment and domain wall motions.

Figure 4c presents the loss tangent of the PVDF‐TrFE and AGO nanocomposites in the frequency range from 300 Hz to 5 MHz. Previously, it has been shown that employing a proper nanocomposite strategy can effectively suppress the loss tangent.^[^ ^33^ ^]^ With the 1 wt.% AGO being the exception, all nanocomposite films demonstrated lower dielectric loss compared with pristine PVDF‐TrFE. At 1 kHz the pristine PVDF‐TrFE and the 0.1 wt.% AGO nanocomposite film had a loss tangent of 0.061 and 0.050, respectively. On the other hand, the 0.3 wt.% AGO film demonstrated the lowest loss tangent equal to *tan δ* = 0.040, corresponding to 35% decrease with respect to pristine PVDF‐TrFE. Despite this improvement, the current dielectric loss values are still high for device applications and have to be further reduced to around *tan δ* = 0.003 to prevent device overheat. Figure 4d displays the leakage current density (*J*) measurements. All the samples, except 0.05 wt.%, AGO, had a higher leakage current density than pristine PVDF‐TrFE at relatively high electric fields. This trend was predictable, since filler incorporation introduces new defects into the polymer matrix, which can act as trap sites to facilitate hopping conduction.^[^ ^34^ ^]^ Further studies need to be done to explain the nature of the anomalous leakage current reduction in 0.05 wt.% AGO nanocomposite film. Figure 4e is a schematic depiction of microcapacitor formation within a film capacitor with a dielectric layer comprised of PVDF‐TrFE and AGO, resulting in overall boosting of relative permittivity, as discussed earlier.

### 2.4 Ferroelectric Performance

To be employed in ferroelectric devices, such as low‐power logic and FRAMs, a ferro‐material needs to demonstrate high remnant polarization and low coercive field. To investigate the influence of the AGO incorporation on *P*_r_ and *E*_c_ values of PVDF‐TrFE and the AGO nanocomposite films, bipolar polarization‐electric field (P‐E) loop were measured at different applied electric fields (Figure 5a–g). All the samples showed broad and saturated hysteresis loops at relatively high electric fields, confirming the ferroelectric characteristic of both pristine PVDF‐TrFE and the AGO nanocomposite films. According to the P‐E and I‐E hysteresis loops (Figure S7, Supporting Information), the 0.1 wt.% AGO nanocomposite film started experiencing ferroelectric domain switching at an applied electric field of 666 kV cm^−1^, which is notably lower than that of pristine PVDF‐TrFE (833 kV cm^−1^). In comparison, in the films with 0.7 and 1 wt.% AGO content, the ferroelectric domain switching started taking place at even higher electric fields (1,166 kV cm^−1^). This trend is of great importance, as it can pave the way for fabricating flexible ferroelectric devices with reduced power consumption. Figure 5h demonstrates the *E*_c_ values obtained from P‐E loops at an applied electric field of 1,166 kV cm^−1^. At low filler concentrations, a notable decrease in *E*_c_ was observed with the lowest value for the 0.1 wt.% AGO film with *E*_c_ = 480 kV cm^−1^. Increasing the AGO concentrations above 0.5 wt.% led to a sharp increase in the coercive field values. In general, the influence of AGO on coercivity of PVDF‐TrFE can be explained through two opposing factors. One is by increasing the size of crystallites as confirmed by SAXS data (Figure 3f), which lead to decrease in the coercive field according to Kay and Dunn empirical model (*E*_c_ ∝ *h* ^−2/3^, where h is crystallite thickness);^[^ ^35^ ^]^ and the other is by electrostatic interaction with domains that causes resistance toward their reorientation, thus increasing *E*_c_. As can be observed in Figure 5i, at low filler concentrations, the first factor is dominant, resulting in an overall decrease in *E*_c_, while, at higher filler concentrations, the electrostatic forces outweigh, thus, increase the *E*_c_ drastically.

Figure S8 (Supporting Information) demonstrates the magnitude of *P*_r_ for pristine PVDF‐TrFE and the AGO nanocomposites as function of applied electric field. As can be seen, *P*_r_ is field‐dependent, inferring that the highest achievable remnant polarization in the samples is influenced by their *E*_b_ The nanocomposite film with 0.1 wt.% AGO demonstrated a promising maximum remnant polarization of 11.3 µC cm^−2^ at 2450 kV cm^−1^ (Figure 5i). This value is one of the highest reported *P*_r_ values for PVDF‐TrFE with a non‐ferroelectric filler. It is noteworthy to mention that this was achieved with only a small quantity of filler and without employment of expensive processing routes such as electrospinning, making it suitable to scale up. The trend in the *P*_r_ values is identical to the *L*_p_ trend (Figure 3f), indicating that the remnant polarization is also influenced by the crystallite size, where bigger crystallites retain more *P*_r_.

### 2.5 Piezoelectric Performance

**Figure 6:**
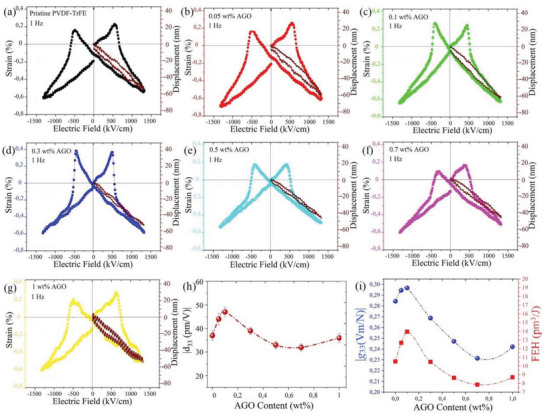
Piezoelectric characterization of PVDF‐TrFE and the AGO nanocomposites. Bipolar and unipolar S‐E hysteresis loops of a) pristine PVDF‐TrFE, b) 0.05 wt.% AGO nanocomposite, c) 0.1 wt.% AGO nanocomposite, d) 0.3 wt.% AGO nanocomposite, e) 0.5 wt.% AGO nanocomposite, f) 0.7 wt.% AGO nanocomposite, and g) 1 wt.% AGO nanocomposite. h) Longitudinal piezoelectric coefficient of pristine PVDF‐TrFE and the AGO nanocomposites. i) The g_33_ and FEH values for pristine PVDF‐TrFE and the AGO nanocomposites.

**Figure 7:**
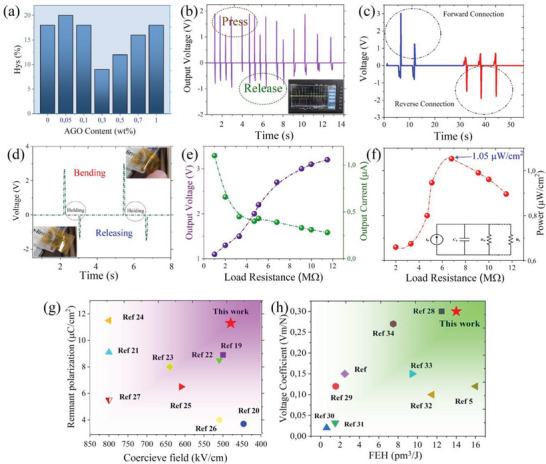
Device performance of interracially engineered PVDF‐TrFE. a) Piezoelectric hysteresis values obtained from unipolar strain loops. Open‐circuit voltage output b) during press and release by finger taps, c) in opposite bias connections, and d) during bending stresses. e) The output voltage and current signals as function of load resistance. f) The instantaneous harvested power density as a result of finger tap. Comparative plots for g) ferroelectric and h) piezoelectric performance of 0.1 wt.% AGO nanocomposite with state‐of‐the‐art reports.

Piezoelectricity is by far the most investigated and sought‐after electrical property of PVDF‐based polymers with the purpose of finding a flexible and biocompatible alternative for PZT. A high piezoelectric coefficient along with a low strain hysteresis are two important requirements for the materials to be used in piezoelectric devices. To investigate the piezoelectric properties of PVDF‐TrFE and the AGO nanocomposite films, bipolar and unipolar strain hysteresis measurements were carried out (**Figure** **6**a–g). All the samples demonstrated reverse butterfly bipolar hysteresis loop, which is characteristic of the piezoelectric response in ferroelectric materials with a negative longitudinal charge coefficient (*d*_33_). The magnitude of hysteresis initially increased with increasing the electric field amplitude and reached its maximum value at electric fields close to the *E*_c_, and then gradually decreased by further increase in the applied electric field. The longitudinal piezoelectric coefficient of the films was measured from the slope of unipolar S‐E hysteresis loops based on the converse piezoelectric effect (Figure 6h). At low filler concentrations, a significant improvement in *d*_33_ values was observed with the highest piezoelectric coefficient of −47 pm V^−1^ for the 0.1 wt.% AGO nanocomposite film, which was ≈27% higher than that of the pristine PVDF‐TrFE. The trend in *d*_33_ values follows the same fashion as in the *β* phase content of the samples demonstrated in Figure 3d. This trend is predictable and consistent with the current understanding of the nature of piezoelectricity in PVDF‐based materials, where non‐polar *α* and amorphous phases have no contribution to the piezoelectricity.^[^ ^36^ ^]^ Assuming that the obtained converse piezoelectric charge coefficient [*d*_33_, pm V^−1^] values are equal to the direct piezoelectric charge coefficient [*d*_33_, pC N^−1^], the piezoelectric voltage coefficient (*g*_33_ = *d*_33_/*ɛ_o_ɛ*_r_) and figure of merit for energy harvesting (FEH = *d*_33_.*g*_33_) values were calculated (Figure 6i). As expected, the 0.1 wt.% AGO nanocomposite film demonstrated the optimum results with |*g*_33_| and FEH values equal to 0.30 Vm N^−1^ and 14 pm^3^ J^−1^, respectively. The obtained |*g*_33_| value is around one order of magnitude higher than the existing commercial piezoceramics such as PZT and BTO with *g*_33_ ≈ 0.02–0.04 Vm N^−1^,^[^ ^37^ ^]^ making it a promising candidate for sensor applications. In terms of energy harvesting performance, the 0.1 wt.% AGO is also among the best reported nanocomposite materials in the literature up to our best knowledge.^[^ ^37^, ^38^ ^]^ The hysteresis percentage (*Hys*%) for pristine PVDF‐TrFE and the AGO nanocomposite films was obtained by dividing the maximum strain difference (Δ*S*_max_) to the maximum obtained strain (*S*_max_) in unipolar S‐E loops (**Figure** **7**a). As seen, all the samples demonstrated *Hys*% ≤ 20%, with the lowest *Hys* = 9% for the 0.3 wt.% AGO nanocomposite. This value indicates 50% improvement in *Hys*% compared to pristine PVDF‐TrFE with *Hys* = 18%. **Table** **1** summarizes the electrical performance of PVDF‐TrFE and the AGO nanocomposites.

**Table 1:** Electrical performance of PVDF‐TrFE and AGO nanocomposites

Sample	*ε*_**r**_ [1 kHz]	*Tan δ* [1 kHz]	*U*_max_ [J cm^−3^]	*P*_r_ [µC cm^−2^]	*E*_c_ [kV cm^−1^]	|*d*_33_| [pm V^−1^]	*g*_33_ [Vm N^−1^]	FEH [pm^3^ J^−1^]	*Hys* [%]
Pristine PVDF‐TrFE	14.7	0.060	3.29	8.4	505	37	0.2820	10.52	18
0.05 wt.% AGO	16.8	0.049	4.03	8.4	493	44	0.2942	12.66	20
0.1 wt.% AGO	17.9	0.051	4.75	11.3	480	47	0.2991	13.97	18
0.3 wt.% AGO	16.4	0.040	3.06	8.1	515	39	0.2687	10.47	9
0.5 wt.% AGO	16.0	0.050	3.14	10.8	529	33	0.2471	8.65	12
0.7 wt.% AGO	16.8	0.043	3.,91	9.1	597	32	0.2314	7.86	16
1 wt.% AGO	16.5	0.121	2.95	9.9	655	36	0.2421	8.71	18

### 2.6 Device Performance

To investigate the device performance of synthesized nanocomposite film, the output piezoelectric response of 0.1 wt.% AGO nanocomposite was studied at different conditions. The pressure sensing performance was investigated by repeated finger taps with approximate pressure value of 10–20 kPa on the piezoelectric device. As shown in Figure 7b, the device demonstrated good sensitivity to finger impact, which results in voltage output of 1–2 V varying based on tap momentum. The generated piezoelectric signals were comprised of positive and negative pulses. When PVDF‐TrFE nanocomposite is subjected to mechanical stress, the piezoelctric field is generated across its thickness, which causes the positive voltage signal. When the stress is removed, the reverse field causes the electron flow in reverse direction and generates the negative pulse. Consequently, changing the positive and negative connections lead to piezoelectric signal revesal as shown in Figure 7c. The positive piezoelectric pulse is mainly used for energy harvesting applications, while the negative pulse is used for sensing purposes. The same concept is valid for applying strain to a piezoelectric device. Bending stresses generate positive pulse, whose magnitude is proportional to the bending angle. Releasing the sensor to its initial position generates the negative piezoelectric pulse as shown in Figure 7d. As observed, holding the sensor in bending condition does not generate any signal. To investigate power harvesting performance of interfacially engineered PVDF‐TrFE, the output voltage and current signals were recorded as function of load resistance ranging from 1 to 12 MΩ (Figure 7e). It was observed that the output voltage gradually increased with increasing the load resistance and got almost saturated at ≈9 MΩ. On the other hand, the current signal decreased with increasing the load resistance and got saturated at ≈4 MΩ. The instantaneous harvested power density as a result of finger tap is demonstrated in Figure 7f. The maximum power density was achieved at load resistance of 6.8 MΩ, which was equal to 1.05 µW cm^−2^. Figure 7g,h compares the ferroelectric and piezoelectric performance of 0.1 wt.% AGO film with state‐of‐the art reports in the literature.

## 3 Conclusion

In this work, we demonstrated that interfacial engineering of PVDF‐TrFE with amine‐functionalized graphene oxide significantly improved dielectric, ferroelectric, and piezoelectric performance of the fluoropolymer. Briefly, the film with 0.1 wt.% AGO demonstrated an outstanding electrical and electromechanical performance with *P*_r_ = 11.3 µC cm^−2^, *g*_33_ = 0.30 Vm N^−1^, FEH = 14 pm^3^ J^−1^, *E*_c_ = 480 kV cm^−1^, and *U*_max_ = 4.75 J cm^−3^, making it one of the best ceramic‐free nanocomposites reported so far. The nanocomposite film with 0.3 wt.% AGO was superior in terms of loss tangent and hysteresis behavior with *tan δ* = 0.040 and *Hys* = 9%. It was observed that the presence of AGO led to an increase in the *β*/*α* phase ratio in PVDF‐TrFE due to the highly negative zeta potential emanating from presence of primary amines. Furthermore, the AGO incorporation resulted in the increase of crystallite size, which brings about higher magnitude of remnant polarization and lower coercive fields. We believe this work can open up new insights toward structural and morphological tailoring of PVDF‐based materials to enhance their electrical performance and pave the way for their deployment in next‐generation wearable electronics and human‐machine interfaces.^[^ ^39^ ^]^

## 4 Experimental Section

### AGO Synthesis

GO was synthesized with the method developed by Marcano et al.^[^ ^40^ ^]^ Briefly, a 9:1 mixture of concentrated H_2_SO_4_ (95–97%, Merck, Germany) and H_3_PO_4_ (85%, VWR, France) (120:13.3 mL) was slowly added to a mixture of 1 g graphite powder (99%, Alfa Aeser, USA) and 6 g KMnO_4_ (99%, Sigma Aldrich, Germany). After 30 min mixing at room temperature, the reaction mixture was heated up to 50 °C and stirred for 12 h. Then it was cooled down to room temperature and poured into 130 mg ice followed by addition of 2.5 mL aqueous H_2_O_2_ (33%, VWR, France). The resulting light‐brown solution was centrifuged for 10 min at 500 rpm to remove large particles. The precipitate was disposed, and the supernatant was centrifuged again at 7,000 rpm for 1 h. The precipitate was successively washed with 1 m HCl (Sigma Aldrich, Germany), 2×Milli‐Q water, and ethanol (96%, Sigma Aldrich, Germany). The obtained product was freeze‐dried overnight, obtaining ≈0.5 g GO. AGO was synthesized by reacting ammonium hydroxide with the obtained GO. Here, 0.1 g GO was ultrasonically dispersed in 80 mL *N*, *N*‐dimethylformamide (DMF) (99.8%, Sigma Aldrich, Germany) followed by addition of 0.2 mL ammonium hydroxide solution (25%, Sigma Aldrich, Germany). The mixture was stirred at room temperature overnight until its light‐brown color turned dark brown (Figure S1a, Supporting Information). The mixture was centrifuged at the speed of 7700 rpm for 12 h, and the precipitate was vacuum dried. The obtained product was washed with Milli‐Q water and vacuum dried overnight.

### Preparation of PVDF‐TrFE Nanocomposite Films

PVDF‐TrFE (1.5 g) (70 mol% VDF and 30 mol% TrFE) powder (Sigma Aldrich, France) was dissolved in 50 mL DMF at 60 °C to form a 30 mg mL^−1^ solution. AGO dispersion with concentration of 1.25 mg mL^−1^ was prepared by overnight reaction of 100 mg GO with 0.2 mL ammonium hydroxide solution (25%) in 80 mL DMF. Note that the drying step was skipped since dried AGO is not dispersible in DMF. The proper amount of PVDF‐TrFE solution and AGO dispersions was mixed to form nanocomposite solutions with AGO content of 0.00, 0.05, 0.1, 0.3, 0.5, 0.7, and 1 wt.%. The obtained solutions were then casted on microscope glass slides and dried at 50 °C to produce films with thickness of ≈12 µm. The films were thermally annealed at 140 °C for 2 h (Figure S1b, Supporting Information).

### Material Characterization

Infrared spectra were collected using a FT‐IR spectrometer (Thermo‐Scientific, iS50, USA) equipped with a ZnSe ATR crystal (iD5, Thermo Scientific, USA) with scanning resolution of 1 cm^−1^ (10 scans). The phase structure of GO and AGO was studied by powder XRD Aeris Research edition instrument (Malvern Panalytical Ltd., Malvern, UK) equipped with a Cu anode X‐ray source. Raman spectroscopy was performed using an inVia confocal Raman microscope (Renishaw, UK) equipped with a 300 mW, 514 nm laser, operating with an intensity of 50 mW and spectral resolution of 1 cm^−1^. Bright field transmission electron microscopy (TEM) images were acquired by TALOS F200A (Thermo Fisher, USA) transmission electron microscope equipped with a TWIN lens system, an X‐FEG electron source and a Ceta 16 m Camera at 200 kV. The AGO dispersion with concentration of 1.25 mg mL^−1^ was diluted with DMF/absolute ethanol (≈1:10 ratio) by sonication (10 min). Five drops of the suspension were drop casted onto a lacey carbon supported Cu grid (300 mesh). The modified grids were dried in vacuum for 2 h before analysis.

The AFM analysis was performed on a Bruker Dimension AFM (Bruker, USA) in contact mode with a platinum‐iridium coated silicon tip (SCM‐PIT‐V2, Bruker, USA). DSC measurements (DSC 3 Mettler Toledo, USA) were carried out to investigate the degree of crystallinity. The samples were placed in aluminum crucibles and heated from 30 to 160 °C with heating rate of 10 °C min^−1^ under nitrogen gas flow. The total degree of crystallinity was calculated from Equation 2.

(2) $$\chi_{c}=\frac{\Delta H_{m}}{\Delta H_{0}}\times 100$$

In this expression, Δ*H*_m_ is obtained by integrating the area under the melting peak located at around 144 °C, and Δ*H*_0_ is the melting enthalpy for fully crystalline PVDF‐TrFE (45 J g^−1^).^[^ ^41^ ^]^ The scanning electron microscopy (SEM) images were collected by a CLARA UHR SEM (TESCAN, Czech Republic), equipped with an Everhart–Thornley type (YAG Crystal) detector for secondary electron imaging. The four quadrant back scattering electron detector was a Retractable Low Energy – 4Q Backscatter electron detector. A Kratos Axis Ultra‐DLD spectrometer (Kratos Analytical Ltd, UK) with a monochromatic Al K‐alpha X‐ray source at power = 150 W was used for XPS analysis. Survey spectra for both GO and AGO were obtained by two sweeps from 0 to 1,600 eV at a pass energy of 160 eV and chamber pressure of 5 × 10^−9^ mbar. Charge correction was done using C1s high‐resolution peak with binding energy of 284.5 eV. Small angle X‐ray scattering (SAXS) and wide‐angle X‐ray scattering (WAXS) measurements were carried out using Nano‐inXider (Xenocs, France) equipped with Genix3D micro‐focus X‐ray Cu K*α* source with 8.04 KeV energy and 0.154 nm wavelength. 2D hybrid pixel detectors of Pilatus 100K and Pilatus 200K (Detrics, Switzerland) were used for WAXS and SAXS, respectively. Relative amount of *α* and *β* crystalline phases in PVDF‐TrFE and AGO nanocomposite films was obtained from 1D‐radial profiles extracted from 2D‐WAXS images. After background subtraction, all profiles were deconvoluted into five peaks at scattering vectors of 1.37, 1.41, 2.48, 2.78, and 2.86 Å^−1^, corresponding to (110)*α*, (110)(200)*β*_ter_, (001)*β*, (002)*α*, and (201)(111)*β*, respectively. A proportion of each crystalline phase was obtained by integrating the area under its corresponding peaks and dividing to total area and then multiplying to total crystalline content obtained from DSC. To achieve structural features, the 2D‐SAXS patterns were azimuthally integrated to obtain 1D‐SAXS profiles.^[^ ^42^ ^]^ To obtain information about lamellae dimensions and nano‐structures of the samples, electron density auto‐correlation function of density fluctuation was calculated according to the following procedure using SasView 5.05 software.

The raw data were first extrapolated from *q* = 0.01 Å^−1^ to 0 by fitting a Guinier function (Equation 3).

(3) $$I\left(q\right)\;=\;Ae^{Bq^{2}}$$

where *A* is the Guinier constant, and *B* is related to effective radius of gyration of a spherical object with same SAXS profile. Extrapolation from *q* = 0.1 Å^−1^ to □ was done by using a Porod model (Equation 4)

(4) $$I(q)=Kq^{-4}e^{-q^{2}\sigma^{2}}+\mathrm{Bg}$$

where *K* is the Porod constant, *Bg* is the background, and *σ* is related to the width of the electron scattering length density profile at the crystalline‐amorphous interfaces. The extrapolated data and original data were then smoothed together using the algorithm in Equation 5.

(5) $$y\left(x_{i}\right)=h_{i}g\left(x_{i}\right)+\left(1-h_{i}\right)f\left(x_{i}\right)$$

where h_i_ = $\frac{1}{1+\;\frac{{\left(x_{i}-b\right)}^{2}}{{\left(x_{i}-a\right)}^{2}}}$

Finally, Fourier transform was applied to the smoothed data [*I*(*q*)] to calculate the correlation function as in Equation 6.

(6) $$K\left(r\right)\;=\frac{1}{Q^{*}}\int_{0}^{\infty }\;I\left(q\right)q^{2}\cos\left(qx\right)dq$$

### Electrical Measurements

Dielectric measurements were accomplished on the Ag/PVDF‐TrFE nanocomposite/Ag capacitor configuration using E4990A impedance analyzer (Keysight Technologies, USA) within the frequency range of 300 Hz–5 MHz at room temperature. Ferroelectric, piezoelectric, leakage current, and I‐E measurements were done with a TF1000 (aixACCT, Germany) equipped with a TFSHU thin film sample holder (aixACCT, Germany). The measurements were executed with an Ag/PVDF‐TrFE nanocomposite/Ag parallel plate capacitor configuration, where Ag electrodes were painted on both sides of the PVDF‐TrFE nanocomposite films. Leakage current measurements were done by applying DC step‐voltage (in both forward and reverse bias) from 0 to 2,000 V with voltage step of 100 V and step duration of 2 s. The P‐E hysteresis loops were acquired by applying electric fields of 666, 833, 1,166, 1,666, and 2,000 kV cm^−1^ with a triangular waveform at 1 Hz. One additional P‐E hysteresis measurement was done at the maximum field strength of each sample to determine the highest achievable remnant polarization. Bipolar and unipolar S‐E loops were obtained by applying an electric field of *E* = 1,350 kV cm^−1^ at 1 Hz. The strain hysteresis percentage was measured from unipolar S‐E loops using the equation *Hys*% = Δ*S*_max_/*S*_max_ × 100, where Δ*S*_max_ is the maximum difference in strain values at a certain electric field, and *S*_max_ is the maximum strain.^[^ ^43^ ^]^ The longitudinal piezoelectric coefficient *d*_33_ (pm V^−1^) of the films was measured from the slope of unipolar S‐E hysteresis loops based on the converse piezoelectric effect.

### Device Performance

Piezoelectric component was fabricated by employing 0.1 wt.% AGO nanocomposite as active layer. The electrodes were painted on both sides of the film using silver nanoparticle inks (JS‐A211, Novacentrix, USA) with the area of 1 × 1 cm^2^ followed by overnight thermal annealing at 80 °C in a vacuum oven.^[^ ^44^ ^]^ Then the wires were integrated to the nanocomposite film and encapsulated using Kapton tape. The film was poled at 1,500 kV cm^−1^ using TF1000 (aixACCT, Germany) equipped with a TFSHU thin film sample holder (aixACCT, Germany). The voltage signals were acquired using an Oscilloscope (Keysight DSOX 1204 A, USA). The bending measurement was done by connecting the fabricated sensor to a fixed ruler (as shown in Figure 7d). The sensor was bent with a fingertip to a radius of ≈70° with respect to the ruler. The energy harvesting performance, the output voltage, and current signals were recorded as function of load resistance ranging from 1 to 12 MΩ.

## Conflict of Interest

The authors declare no conflict of interest.

## Supporting information

Supporting Information

## Data Availability

The data that support the findings of this study are available from the corresponding author upon reasonable request.
