# Supplementary material for: Interfacial Engineering of PVDF‐TrFE toward Higher Piezoelectric, Ferroelectric, and Dielectric Performance for Sensing and Energy Harvesting Applications
Source: Adv Sci (Weinh). 2023 Jan 3;10(6):2205942. doi: 10.1002/advs.202205942 (PMC9951327; doi:10.1002/advs.202205942)
Supplement: Supplementary file 1 — Supporting Information [file ADVS-10-2205942-s001.pdf]

ADVANCED SCIENCE
Open Access

Supporting Information

for *Adv. Sci.*, DOI 10.1002/advs.202205942

Interfacial Engineering of PVDF-TrFE toward Higher Piezoelectric, Ferroelectric, and Dielectric Performance for Sensing and Energy Harvesting Applications

*Hamed Abdolmaleki, Astri Bjørnetun Haugen, Kristian Birk Buhl, Kim Daasbjerg and Shweta Agarwala**

Supporting Information

Interfacial Engineering of PVDF-TrFE towards Higher Piezoelectric, Ferroelectric, and Dielectric Performance for Sensing and Energy Harvesting Applications

Hamed Abdolmaleki[1], Astri Bjørnetun Haugen[2], Kristian Birk Buhl[3], Kim Daasbjerg[4], Shweta Agarwala[1]*

1- *Department of Electrical and Computer Engineering. Aarhus University. Denmark*
2- *Department of Energy Conversion and Storage. Technical University of Denmark (DTU). Denmark*
3- *Danish Graphene ApS. Vejle. Denmark*
4- *Novo Nordisk Foundation (NNF) Research Center, Department of Chemistry and Interdisciplinary Nanoscience Center (iNANO), Aarhus University, Denmark*

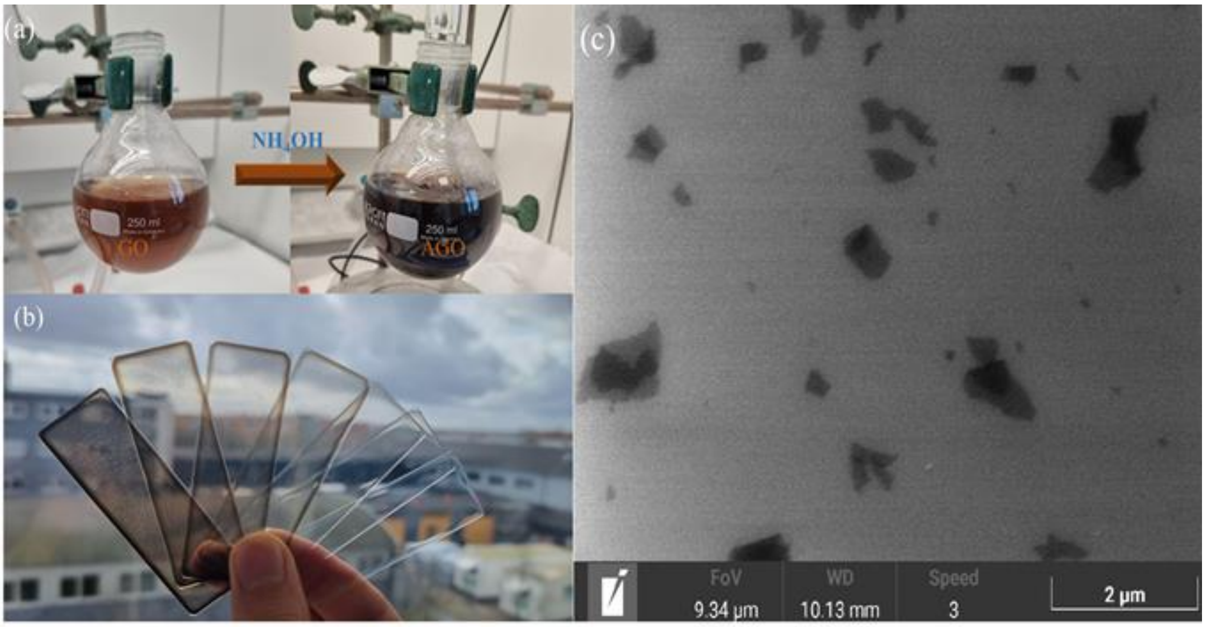

Supporting Fig. 1. a Color change of GO dispersion from light brown to dark brown after addition of ammonium hydroxide, which corroborates AGO formation. **b** Pristine PVDF-TrFE and AGO nanocomposite films with various AGO content ranging from 0.05 to 1 wt%. **c** SEM image of AGO nanosheets.

Table S1. Elemental Composition of GO and AGO Obtained from XPS

	GO				AGO			
	C1s (%)	O1s (%)	S 2p (%)	Si 2p (%)	C1s (%)	O1s (%)	N1s (%)	S 2p (%)
Spot 1	25.9	72.2	1.0	0.8	30.6	64.7	3.6	0.9
Spot 2	27.9	70.9	0.3	0.8	29.9	66.0	3.5	0.5
Spot 3	29.0	69.5	0.8	0.6	29.2	66.7	3.6	0.5
Spot 4	27.	71.4	0.5	0.6	27.8	68.6	3.0	0.5
Spot 5	27.9	70.8	0.7	0.8	27.5	68.6	3.6	0.2
Spot 6	25.7	72.9	0.7	0.7	27.3	69.8	2.7	0.2
Average	27.3	71.3	0.7	0.7	28.7	67.4	3.4	0.5
Std Dev	1.1	1.1	0.2	0.1	1.3	1.7	0.4	0.2

Table S2. Obtained Data from Deconvolution of High-resolution C1s Spectrum of GO

GO	C-C/C=C		C-OH/C-O-C		C=O/O-C=O	
	Position	%At Conc	Position	%At Conc	Position	%At Conc
Spot 1	284.4	55.8	286.8	37.5	288.6	6.7
Spot 2	284.6	50.4	287.0	41.2	288.8	8.4
Spot 3	284.5	46.6	286.9	43.8	288.6	9.6
Spot 4	284.7	51.4	287.1	39.0	288.8	9.5
Spot 5	284.7	54.5	287.0	36.8	288.8	8.6
Spot 6	284.7	57.3	287.0	34.9	288.9	7.7
Average	284.6	52.7	287.0	38.9	288.8	8.4
Std Dev	0.1	3.6	0.1	2.9	0.1	1.0

Table S3. Obtained Data from Deconvolution of High-resolution C1s Spectrum of AGO

AGO	C-C/C=C		C-OH/C-O-C/C-N		C=O/O-C=O		***O-C=O$^{(-)}$***	
	Position	%At Conc	Position	%At Conc	Position	%At Conc	Position	%At Conc
Spot 1	284.3	44.8	286.4	38.6	287.8	14.8	290.2	1.7
Spot 2	284.5	46.6	286.6	37.2	288.0	13.4	290.0	2.8
Spot 3	284.3	43.1	286.4	41.0	287.9	12.2	289.8	3.6
Spot 4	284.3	48.6	286.5	37.9	288.1	10.8	289.9	2.6
Spot 5	284.6	50.6	286.7	33.9	288.2	14.3	291.0	1.6
Spot 6	284.5	50.0	286.7	33.5	288.1	14.7	291.0	1.7
Average	284.4	47.3	286.6	37.0	288.0	13.4	290.3	2.3
Std Dev	0.1	2.7	0.1	2.7	0.1	1.5	0.5	0.7

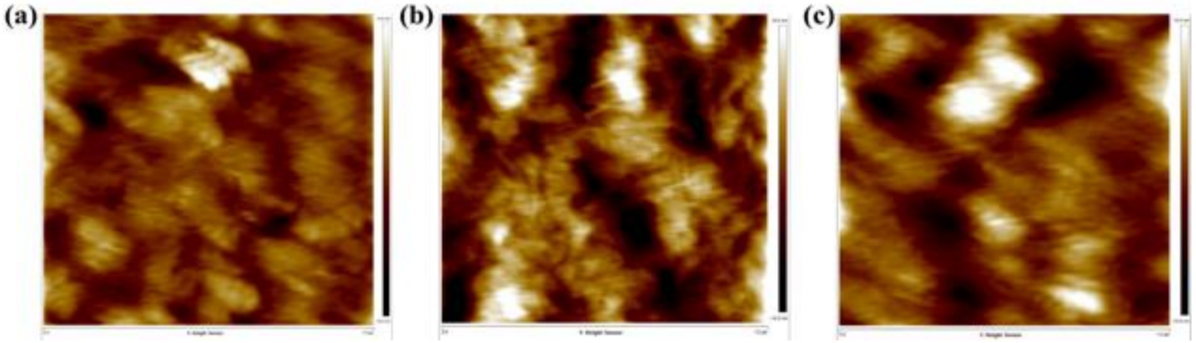

Supporting Fig. 2. AFM images of **a** pristine PVDF-TrFE, **b** 0.1 wt% AGO nanocomposite film, and **c** 1 wt% AGO nanocomposite film.

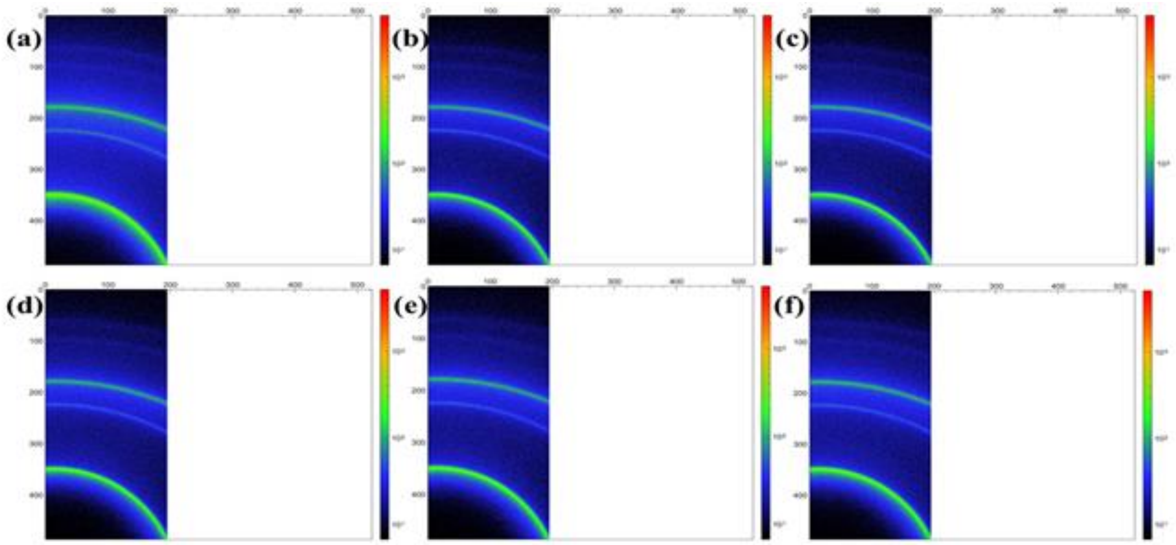

Supporting Fig. 3. 2D-WAXS scattering patterns of nanocomposite films with AGO content of **a** 0.05, **b** 0.1, **c** 0.3, **d** 0.5, **e** 0.7, and **f** 1 (wt %).

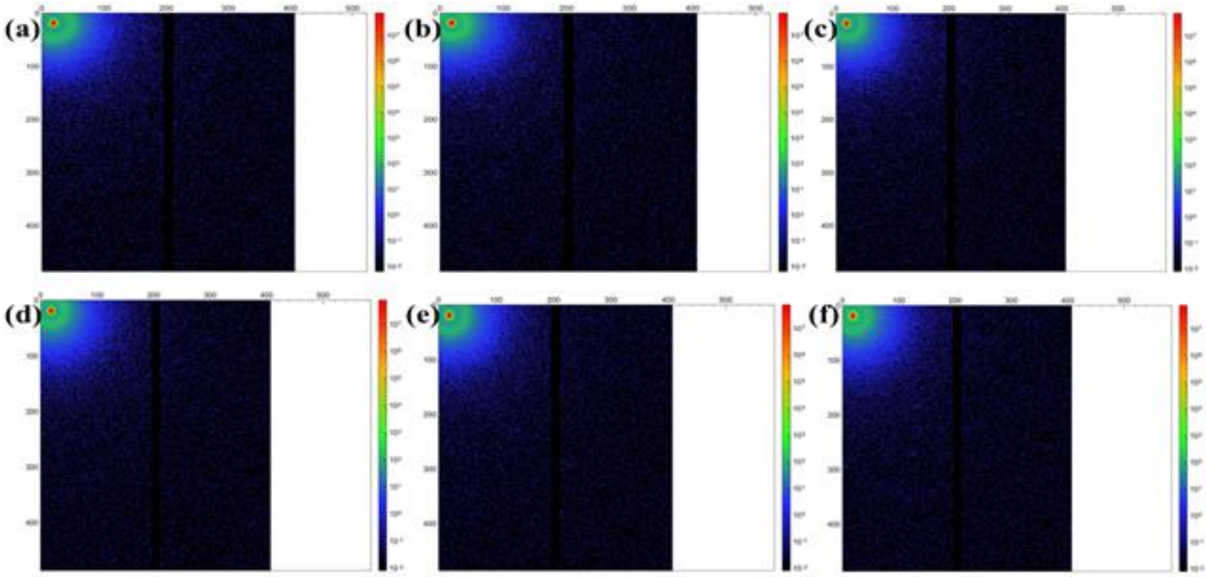

Supporting Fig. 4. 2D-SAXS scattering patterns of nanocomposite films with AGO content of **a** 0.05, **b** 0.1, **c** 0.3, **d** 0.5, **e** 0.7, and **f** 1 (wt %).

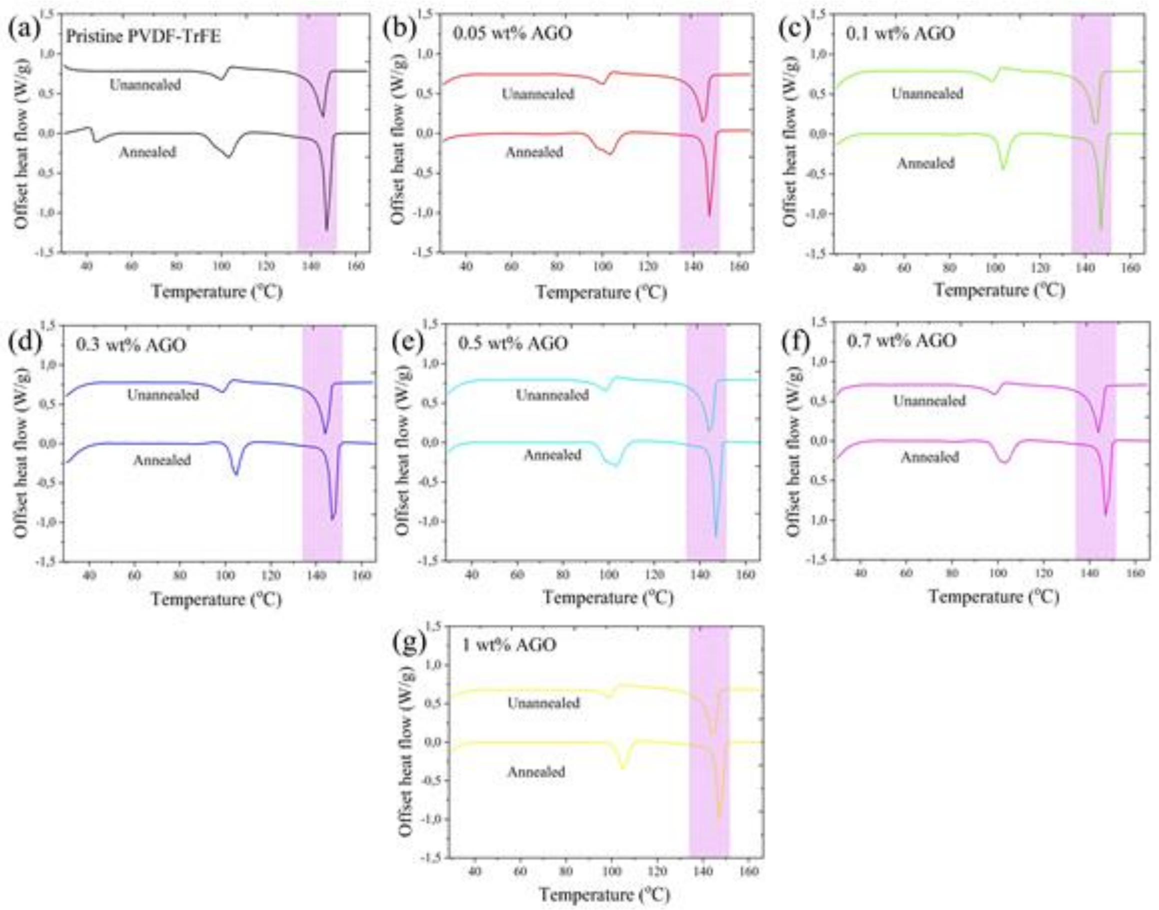

Supporting Fig. 5. Differential scanning calorimetry (DSC) plots for annealed and unanneald films for **a** pristine PVDF-TrFE, and nanocomposite films with AGO content of **b** 0.05, **c** 0.1, **d** 0.3, **e** 0.5, **f** 0.7, and **g** 1 (wt%).

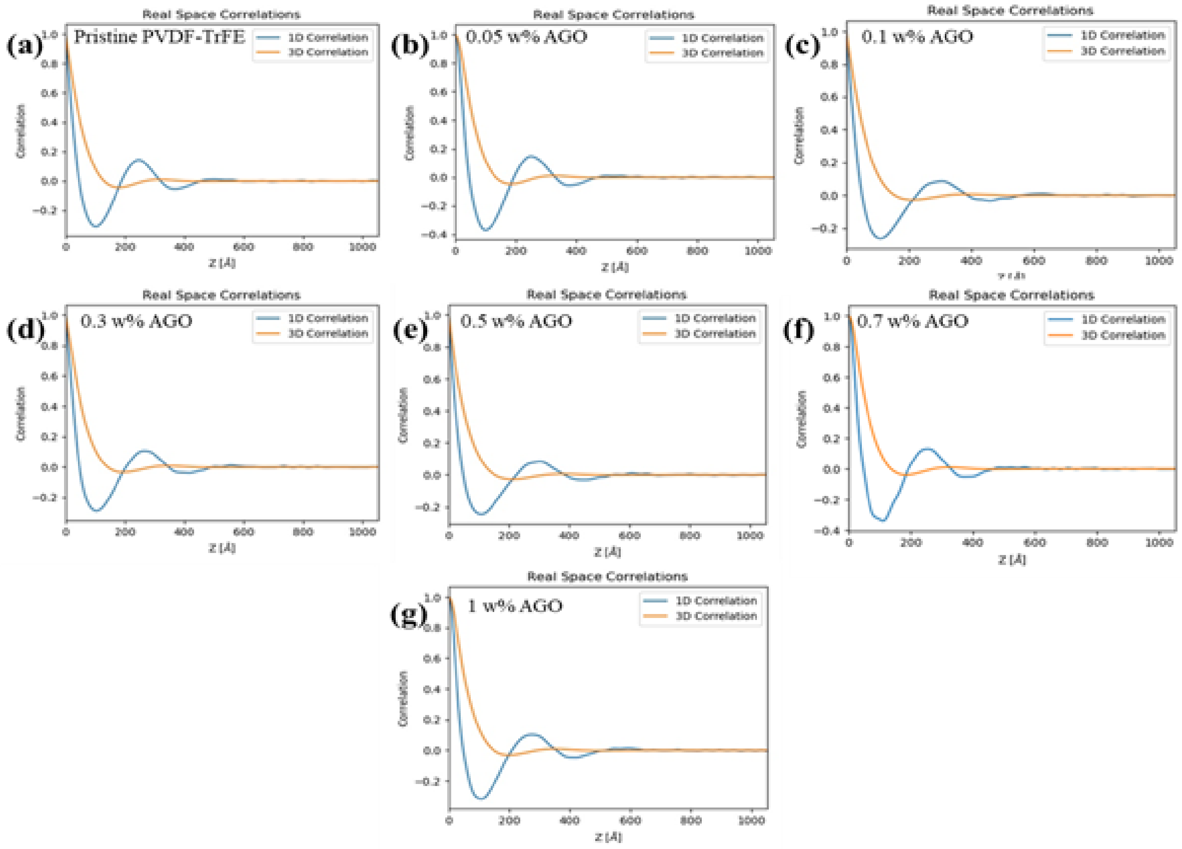

Supporting Fig. 6. Electron density auto-correlation functions of density fluctuation for **a** pristine PVDF-TrFE, and nanocomposite films with AGO content of **b** 0.05, **c** 0.1, **d** 0.3, **e** 0.5, **f** 0.7, and **g** 1 (wt%).

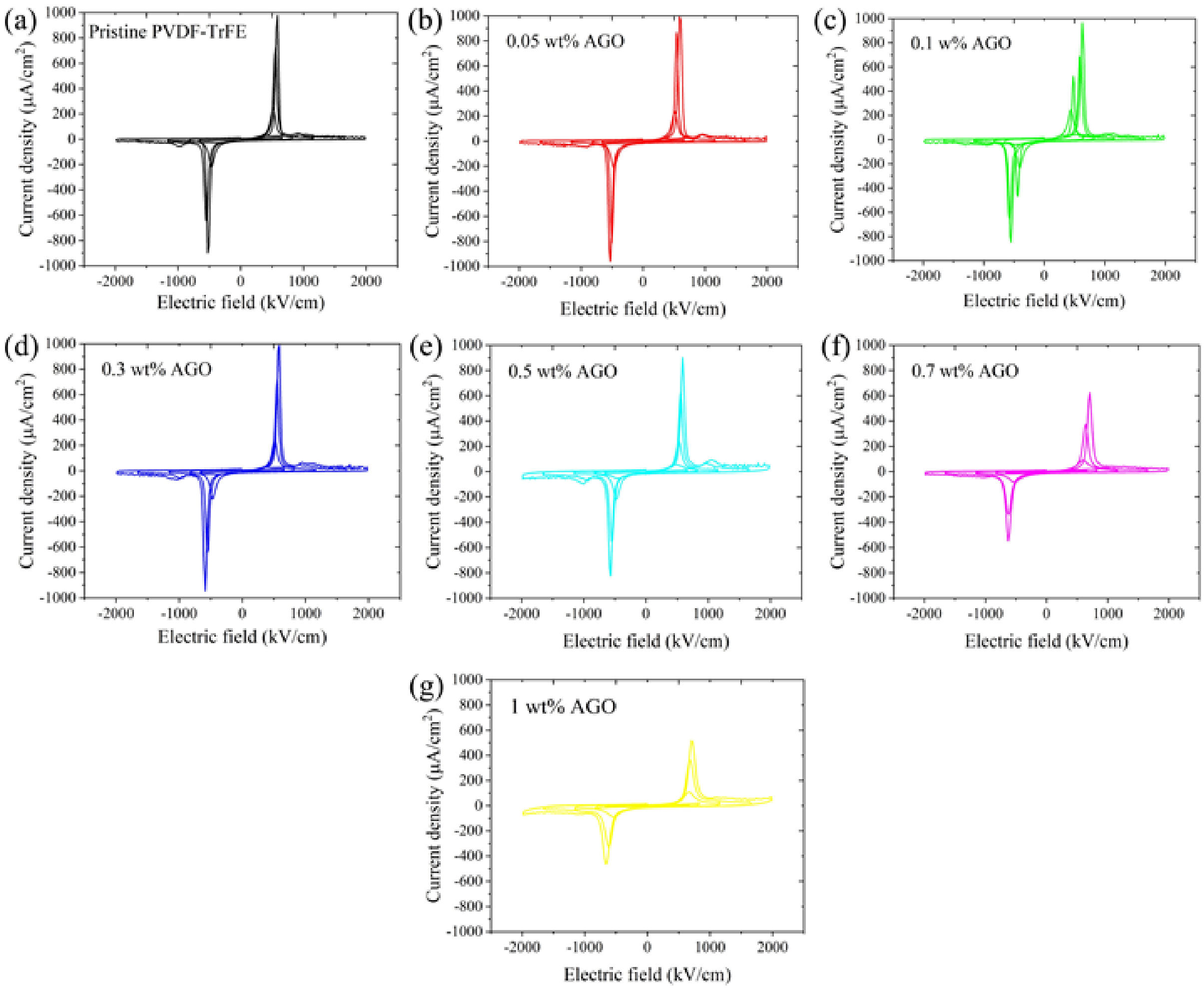

Supporting Fig. 7. Ferroelectric domain switching E-V plots for **a** pristine PVDF-TrFE, and nanocomposite films with AGO content of **b** 0.05, **c** 0.1, **d** 0.3, **e** 0.5, **f** 0.7, and **g** 1 (wt%).

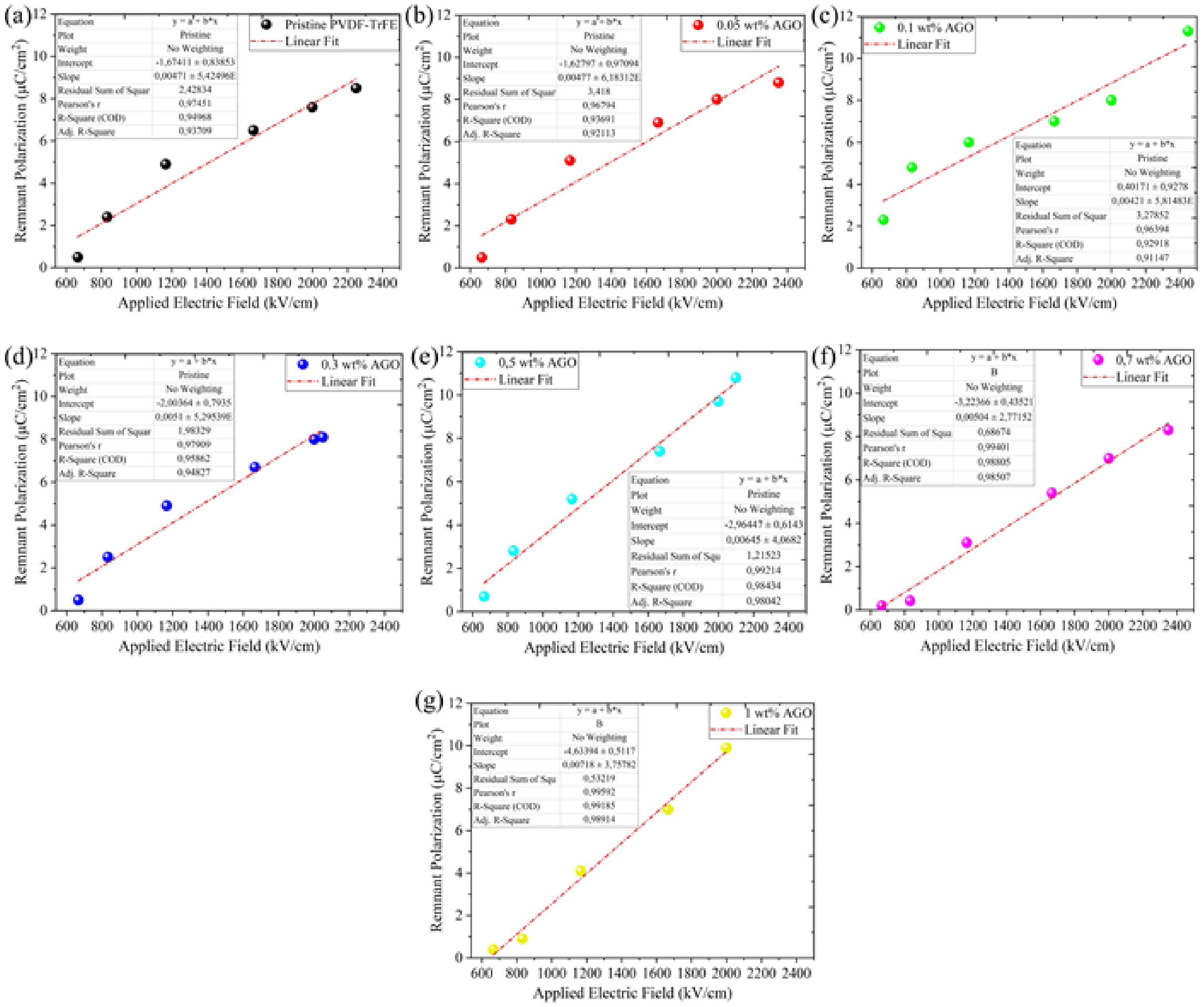

Supporting Fig. 8. Remnant polarization values obtained at applied electric fields of 666, 833, 1166, 1666, 2000, and the maximum field strength (kV/cm) for **a** pristine PVDF-TrFE, and nanocomposite films with AGO content of **b** 0.05, **c** 0.1, **d** 0.3, **e** 0.5, **f** 0.7, and **g** 1 (wt%).
